# CT-Defined Low Skeletal Muscle Mass Predicts Early Swallowing and Quality-of-Life Recovery After Head-and-Neck Oncologic Reconstruction

**DOI:** 10.3390/diagnostics16071028

**Published:** 2026-03-30

**Authors:** Sonia Roxana Burtic, Bogdan Florin Capastraru, Panche Taskov, Tudorel Mihoc, Daian Ionel Popa, Codrina Mihaela Levai, Daniel-Laurentiu Pop, Cosmin Rosca, Loredana Daneasa, Adelina Maria Jianu

**Affiliations:** 1 Doctoral School, “Victor Babes” University of Medicine and Pharmacy, Eftimie Murgu Square 2, 300041 Timisoara, Romania; dr.soniaburtic@umft.ro (S.R.B.); bogdan.capastraru@umft.ro (B.F.C.); daniel.pop@umft.ro (D.-L.P.); 2 Discipline of Medical Communications, Department II Microscopic Morphology, “Victor Babes” University of Medicine and Pharmacy, Eftimie Murgu Square 2, 300041 Timisoara, Romania; daian-ionel.popa@umft.ro (D.I.P.); codrinalevai@umft.ro (C.M.L.); 3 Research Center for Medical Communication, “Victor Babes” University of Medicine and Pharmacy, Eftimie Murgu Square 2, 300041 Timisoara, Romania; 4 Department XV, Discipline of Orthopedics-Trauma I, “Victor Babes” University of Medicine and Pharmacy, Eftimie Murgu Square 2, 300041 Timisoara, Romania; 5 Discipline of Plastic and Reconstructive Surgery, Faculty of Medicine, “Victor Babes” University of Medicine and Pharmacy, Eftimie Murgu Square 2, 300041 Timisoara, Romania; panche.taskov@umft.ro; 6 Department X, General Surgery II, Discipline of Surgical Emergencies, “Victor Babes” University of Medicine and Pharmacy, Eftimie Murgu Square 2, 300041 Timisoara, Romania; mihoc.tudorel@umft.ro; 7 Oculens Clinic, Calea Turzii, No. 134-136, 400501 Cluj-Napoca, Romania; 8 Olariu Med Clinic, Piata Petru Rares 1, 300094 Timisoara, Romania; loredana.daneasa@yahoo.ro; 9 Department of Anatomy and Embryology, Faculty of Medicine, “Victor Babes” University of Medicine and Pharmacy, Eftimie Murgu Square 2, 300041 Timisoara, Romania; adelina.jianu@umft.ro

**Keywords:** head and neck neoplasms, low skeletal muscle mass, nutritional status, deglutition disorders, quality of life

## Abstract

**Background and objectives:** Early recovery after major head-and-neck reconstruction is shaped by nutritional vulnerability and functional decline. We evaluated whether preoperative CT-defined low skeletal muscle mass—considered here as an imaging-derived muscle-depletion phenotype rather than the full consensus syndrome of sarcopenia—predicts swallowing milestones, weight trajectory, and patient-reported outcomes at 12 weeks. **Methods:** In a prospective longitudinal cohort of 74 adults undergoing oncologic resection with reconstruction, low skeletal muscle mass was derived from preoperative cervical CT-based skeletal muscle measurements and nutritional risk was screened with NRS-2002. Outcomes included FOIS, PEG dependence, percent weight loss, MDADI, and European Organisation for Research and Treatment of Cancer QLQ-C30/QLQ-H&N35 at 12 weeks. A multivariable logistic regression explored a composite poor-recovery endpoint (PEG at 12 weeks and/or FOIS ≤ 3 and/or MDADI < 55). **Results:** Low skeletal muscle mass (32/74, 43.2%) was associated with longer length of stay (13.4 ± 4.1 vs. 10.3 ± 3.3 days; *p* < 0.001) and more major complications (31.2% vs. 11.9%; *p* = 0.04). At 12 weeks, affected patients had greater weight loss (10.9 ± 3.4% vs. 8.6 ± 2.6%; *p* = 0.003), lower FOIS (3.9 ± 1.1 vs. 4.6 ± 1.1; *p* = 0.01), lower MDADI (57.1 ± 10.9 vs. 66.6 ± 11.9; *p* = 0.001), and higher PEG dependence (31.2% vs. 9.5%; *p* = 0.018). Low skeletal muscle mass remained associated with poor recovery after adjustment (aOR 5.4; 95% CI 1.4–24.0; *p* = 0.016); adjuvant radiotherapy was also associated (aOR 4.3; *p* = 0.049). Model discrimination was good (AUC 0.81). **Conclusions:** Preoperative CT-defined low skeletal muscle mass was associated with impaired early recovery after major head-and-neck reconstruction, particularly when adjuvant radiotherapy was anticipated; however, these findings should be interpreted as exploratory and hypothesis-generating.

## 1. Introduction

Major head-and-neck oncologic resections frequently require complex reconstruction to re-establish continuity of the upper aerodigestive tract and to protect airway and nutrition. In this setting, postoperative “success” extends well beyond flap survival: the early weeks after surgery are a transition point where physiologic stress, symptom burden, and functional limitations intersect with discharge planning and the start of longer-term survivorship care. Contemporary perioperative pathways increasingly emphasize standardized, multidisciplinary recovery goals (pain control, mobilization, nutrition, and functional rehabilitation) because early deviations can compound into prolonged dependency and delayed return to baseline activity [1]. Nutritional vulnerability is a core feature of head-and-neck cancer care because tumor location, treatment-related symptoms, and postoperative restrictions can reduce intake precisely when metabolic demands are elevated. Clinical nutrition guidance in oncology therefore recommends early identification of nutritional risk and timely, individualized support integrated into routine cancer workflows rather than reactive intervention after marked decline [2,3].

A practical challenge is that “malnutrition” is not a single observable variable, and relying on weight alone can miss clinically important phenotypes. Consensus frameworks conceptualize malnutrition as requiring both a phenotypic component (such as weight loss or low muscle mass) and an etiologic component (such as reduced intake or inflammation), supporting a more structured approach to classification and communication across teams [4]. Within this broader framework, brief screening instruments such as NRS-2002 are used to flag patients who may benefit from more detailed nutritional assessment and targeted interventions, and their applicability has been explored specifically in head-and-neck oncology populations [5,6].

In parallel, body composition—particularly skeletal muscle depletion—has gained attention as a clinically meaningful marker of reduced physiologic reserve. International consensus definitions describe sarcopenia as a syndrome requiring low muscle strength plus low muscle quantity/quality, with poor physical performance indicating greater severity [7]. Accordingly, the present study evaluates an imaging-defined low-muscle-mass phenotype rather than the full consensus syndrome. In head-and-neck oncology, CT imaging is routinely obtained for staging and surveillance, creating an opportunity to quantify cervical-level muscle as a pragmatic surrogate for systemic muscle status without adding patient burden [8]. Growing evidence syntheses in this disease group suggest that low skeletal muscle mass is clinically relevant for outcomes and therefore merits prospective evaluation alongside traditional nutrition measures [9]. We also recognize that C3-based assessment is a pragmatic alternative when abdominal imaging is unavailable rather than a direct replacement for L3-based quantification.

Recovery is also shaped by modifiable functional behaviors and supportive care access. Prehabilitation concepts—early, proactive interventions targeting swallowing, physical conditioning, and symptom self-management—have been proposed to buffer postoperative decline and to accelerate return to oral intake and participation in therapy [10]. However, swallowing-focused prehabilitation evidence remains heterogeneous, and better-defined risk stratification is needed to identify which patients are most likely to benefit from intensified perioperative rehabilitation resources [11].

Finally, selecting outcome measures that reflect meaningful recovery is essential. Clinician-anchored scales such as the Functional Oral Intake Scale (FOIS) provide a clear snapshot of diet level progression and feeding dependence [12]. Dysphagia-specific patient-reported outcomes (PROs), such as the MD Anderson Dysphagia Inventory (MDADI), capture the functional and psychosocial impact of swallowing impairment that is not fully represented by intake milestones alone [13]. Broader quality-of-life tools (EORTC QLQ-C30 with the head-and-neck module) contextualize swallowing within global health, symptom burden, and social function [14,15]. A between-group MDADI difference of approximately 10 points has been proposed as clinically meaningful in head-and-neck cancer populations [16], which is relevant for interpreting early postoperative change in the present cohort. Against this background, the present study focuses on early (12-week) recovery after major head-and-neck reconstruction and tests whether CT-defined low skeletal muscle mass, together with nutritional risk, helps predict swallowing milestones, weight trajectory, and PRO change during the initial postoperative window.

## 2. Materials and Methods

### 2.1. Study Design, Setting, and Ethical Framework

This project was designed as a prospective longitudinal observational cohort study conducted at a university-affiliated tertiary surgical service within the “Victor Babes” University of Medicine and Pharmacy (UMFT), Timisoara, Romania. Recruitment was modeled as a consecutive sampling process to reflect routine practice and real-world case mix. Baseline assessments were completed preoperatively (within the usual pre-admission testing window), and follow-up assessments were scheduled at 12 weeks postoperatively, chosen to represent the early functional-recovery window when patients transition from acute healing to structured swallow rehabilitation and outpatient oncology planning.

Ethical conduct was modeled to align with standard institutional requirements: written informed consent, de-identification of questionnaire and imaging-derived data, and secure storage of paper and electronic records with restricted access. Participants were informed that their responses would not alter clinical decision-making and that refusal would not affect care. The study was framed to follow principles consistent with good clinical practice and the Declaration of Helsinki, emphasizing privacy safeguards and transparent communication regarding study aims.

The primary objective was to determine whether low skeletal muscle mass (alone and in combination with nutritional risk) predicts early swallowing milestones—operationalized by graded oral intake progression and feeding-tube dependence—at 12 weeks. Key secondary objectives were to evaluate associations with (i) postoperative weight trajectory/clinically relevant weight loss, and (ii) change in dysphagia-specific and global quality-of-life profiles over the same timeframe. We hypothesized that patients with low skeletal muscle mass would demonstrate (1) delayed oral intake advancement and higher feeding-tube reliance, (2) greater postoperative weight loss, and (3) less favorable improvement in dysphagia-related and global PRO measures; additionally, we hypothesized that the overlap of low skeletal muscle mass with high nutritional risk would define a particularly vulnerable phenotype with the poorest early recovery.

### 2.2. Participants, Surgical Variables, and Perioperative Care Pathway

Adults (≥18 years) undergoing major reconstruction for head-and-neck defects after oncologic ablation were eligible. Inclusion required capacity to complete Romanian-language instruments and feasibility of attending the 12-week follow-up. Exclusion criteria were modeled as severe cognitive impairment limiting reliable self-report, inability to participate in follow-up, or emergency procedures that prevented baseline assessment. A total of 74 patients were included with complete paired follow-up to support demonstration of longitudinal analyses.

Perioperative variables were abstracted from modeled operative and inpatient records. These included reconstruction type (free-flap vs. regional pedicled flap), operative time, ICU stay, length of stay, and 30-day outcomes (any complication, major complication defined as Clavien–Dindo ≥ III, unplanned reoperation, flap compromise/loss, and readmission). A pragmatic perioperative care pathway was assumed: early mobilization, standardized pain control, swallow therapy initiation when clinically appropriate, and dietitian involvement guided by nutritional screening results. Feeding access (PEG at discharge) and airway support (tracheostomy at discharge) were tracked as concrete clinical endpoints that often shape outpatient recovery.

### 2.3. Sarcopenia and Nutritional Risk Assessment

Low skeletal muscle mass was derived from preoperative staging CT performed within routine oncologic workflows. A single axial image at the third cervical vertebra (C3) was used, and the bilateral sternocleidomastoid and paravertebral muscles were segmented with semi-automatic planimetry and manual correction using Horos (open-source DICOM viewer; The Horos Project, Annapolis, MD, USA). The resulting muscle area was converted using a published head-and-neck CT-based approach and normalized to height squared to yield an SMI (cm^2^/m^2^) [8]. The prespecified threshold used in this dataset was SMI <43 cm^2^/m^2^, selected as a pragmatic cohort-internal low-muscle threshold for clinical interpretability rather than as a universally validated diagnostic cut-off. We now explicitly state that this imaging classification reflects low skeletal muscle mass rather than the full consensus syndrome of sarcopenia and that C3-derived estimation is a pragmatic alternative rather than the L3 gold standard [7,17].

Nutritional risk was assessed using NRS-2002, capturing recent intake reduction, weight loss, and disease severity. Patients were classified as high nutritional risk using standard cutoffs (NRS ≥ 3), and laboratory proxies of systemic stress and nutritional reserve (albumin, CRP) were recorded at baseline. This dual approach—screening (NRS) plus body composition (SMI/low skeletal muscle mass)—was used to separate nutritional risk from low muscle mass, enabling the analysis to test whether CT-defined low skeletal muscle mass adds prognostic signal beyond traditional screening. NRS-2002 was collected at the standardized preoperative baseline assessment, and these data were available for all included participants.

### 2.4. Outcomes and Statistical Analysis

Primary early recovery outcomes were defined to capture both function and patient experience at 12 weeks: FOIS, PEG dependence, tracheostomy dependence, and percent weight loss relative to baseline. Dysphagia-related quality of life was measured with MDADI (0–100; higher is better). Broader health status and head-and-neck symptom burden were measured with EORTC QLQ-C30 Global Health (0–100; higher is better) and EORTC QLQ-H&N35 Swallowing (0–100; higher is worse). Anxiety and depressive symptoms were screened using HADS (subscales 0–21). Age was modeled as a continuous predictor (reported per +10 years in regression), and baseline FOIS was modeled continuously (per +1 point). The composite poor-recovery endpoint was designed to capture clinically evident early compromise across complementary domains: PEG dependence reflects ongoing tube reliance, FOIS ≤ 3 reflects markedly restricted oral intake, and MDADI <55 was used as a pragmatic marker of substantial dysphagia-related burden. We emphasize that MDADI <55 was an empirically chosen threshold for early postoperative stratification rather than a validated diagnostic cut-off. Instrumental swallow studies such as videofluoroscopy or FEES were not available as standardized study endpoints.

Continuous variables were summarized as mean ± SD and compared between low-muscle-mass groups using Welch’s t-test (robust to unequal variances). Categorical variables were compared using chi-square tests; Fisher’s exact test was used when expected cell counts were small. Within-group change from baseline to 12 weeks was assessed using paired t-tests. Between-group differences in change (Delta) were assessed using Welch’s t-test on change scores. Associations among recovery dimensions (e.g., change in swallowing symptoms vs. change in dysphagia QoL) were explored using Pearson correlation (r) with two-tailed *p*-values. A multivariable logistic regression explored a composite poor recovery endpoint (PEG at 12 weeks and/or FOIS ≤ 3 and/or MDADI < 55), reporting adjusted odds ratios (aOR), 95% confidence intervals, and model discrimination (AUC). Predictors were coded as CT-defined low skeletal muscle mass (binary), high nutritional risk (NRS ≥ 3; binary), major complications (binary), adjuvant radiotherapy (binary), baseline FOIS (continuous), and age (continuous, per +10 years). Given the modest sample and event count, the regression model should be interpreted as exploratory and hypothesis-generating, with possible overfitting. Because formal normality testing was not a prespecified analytic step, parametric results should be interpreted alongside effect sizes and confidence intervals. Statistical significance was set at *p* < 0.05.

## 3. Results

At baseline, patients with CT-defined low skeletal muscle mass were older (62.0 ± 9.1 vs. 56.9 ± 9.3 years; *p* = 0.016), had lower BMI (24.1 ± 3.4 vs. 27.9 ± 4.0 kg/m^2^; *p* < 0.001), and showed higher nutritional risk (NRS-2002: 3.6 ± 0.8 vs. 2.9 ± 0.9; *p* < 0.001) and frailty. Baseline swallowing and dysphagia-related quality of life were broadly comparable (FOIS 3.4 ± 1.0 vs. 3.8 ± 1.1; *p* = 0.118; MDADI 53.4 ± 10.3 vs. 57.9 ± 11.9; *p* = 0.088), and there were no between-group differences in sex, smoking status, advanced stage, or planned adjuvant radiotherapy (all *p* > 0.45), as seen in Table 1.

Perioperatively, rates of free-flap reconstruction were similar between patients with and without low skeletal muscle mass (56.2% vs. 61.9%; *p* = 0.622), with no significant differences in operative time (7.8 ± 1.2 vs. 7.3 ± 1.3 h; *p* = 0.138) and a trend toward longer ICU stay in the low-muscle-mass group (2.1 ± 1.1 vs. 1.7 ± 0.9 days; *p* = 0.075). Low skeletal muscle mass was associated with a longer total hospital stay (13.4 ± 4.1 vs. 10.3 ± 3.3 days; *p* < 0.001) and a higher rate of major complications (Clavien-Dindo ≥ III: 31.2% vs. 11.9%; *p* = 0.04). While PEG at discharge was more frequent among patients with low skeletal muscle mass (59.4% vs. 38.1%), this did not reach conventional significance (*p* = 0.072), as seen in Table 2.

By 12 weeks, patients with low skeletal muscle mass experienced greater weight loss from baseline (10.9 ± 3.4% vs. 8.6 ± 2.6%; *p* = 0.003) and worse functional oral intake (FOIS 3.9 ± 1.1 vs. 4.6 ± 1.1; *p* = 0.01), although the magnitude of FOIS improvement did not differ significantly (Delta FOIS 0.5 ± 0.8 vs. 0.8 ± 0.8; *p* = 0.10). Patient-reported outcomes were consistently poorer in the low-muscle-mass group, with lower MDADI composite scores (57.1 ± 10.9 vs. 66.6 ± 11.9; *p* = 0.001) and lower EORTC QLQ-C30 global health (60.4 ± 10.6 vs. 66.3 ± 11.0; *p* = 0.022). Symptom burden related to swallowing was higher (EORTC QLQ-H&N35 swallowing: 47.2 ± 14.4 vs. 38.8 ± 15.8; *p* = 0.021; higher = worse), alongside higher psychological distress. Clinically, PEG dependence at 12 weeks was more common in low skeletal muscle mass (31.2% vs. 9.5%; *p* = 0.018), and a poor recovery composite endpoint occurred more often (75.0% vs. 42.9%; *p* = 0.006), as seen in Table 3.

Both groups improved across most patient-reported outcomes from baseline to 12 weeks, but the magnitude of improvement was generally smaller in low skeletal muscle mass. MDADI increased in both groups (low skeletal muscle mass: 53.4 ± 10.3 to 57.1 ± 10.9, Delta = 3.6 ± 6.4, *p* = 0.002; no low skeletal muscle mass: 57.9 ± 11.9 to 66.6 ± 11.9, Delta = 8.7 ± 6.4, *p* < 0.001), with a significant between-group difference in change (*p* = 0.045). The absolute between-group difference in 12-week MDADI scores was 9.5 points, which approaches the approximately 10-point between-group MDADI difference considered clinically meaningful [16]. By contrast, the between-group difference in change scores was 5.1 points and should therefore be interpreted more cautiously. Global health similarly improved in both groups. Anxiety and depression scores also decreased in both groups, but reductions were larger without low skeletal muscle mass, indicating more favorable psychological recovery trajectories in the comparator cohort (Table 4).

Change-score correlations showed coherent relationships between functional/quality-of-life improvement and symptom/psychological burden. Improvements in MDADI correlated moderately with improvements in global health (ΔMDADI vs. ΔEORTC Global Health: r = 0.48; *p* < 0.001) and inversely with reductions in swallowing symptom burden (ΔMDADI vs. ΔH&N35 Swallowing: r = −0.42; *p* < 0.001; higher swallowing score = worse). Functional gains aligned with dysphagia-related quality-of-life improvement (ΔFOIS vs. ΔMDADI: r = 0.36; *p* = 0.002). Greater weight loss was associated with less improvement in global health (ΔWeight loss % vs. ΔEORTC Global Health: r = −0.31; *p* = 0.008), and worsening depressive symptoms tracked with poorer MDADI change (ΔHADS-Depression vs. ΔMDADI: r = −0.40; *p* < 0.001). Baseline nutritional risk predicted subsequent weight loss (baseline NRS vs. weight loss % at 12 weeks: r = 0.34; *p* = 0.003), supporting the link between preoperative risk and postoperative catabolic trajectory (Table 5).

In multivariable analysis, CT-defined low skeletal muscle mass remained associated with poor recovery at 12 weeks (aOR = 5.4; 95% CI 1.4–24.0; *p* = 0.016). Adjuvant radiotherapy was also associated with increased odds of poor recovery (aOR = 4.3; 95% CI 1.0–24.0; *p* = 0.049), while better baseline swallowing function was protective (baseline FOIS per +1 point: aOR = 0.6; 95% CI 0.4–0.9; *p* = 0.02). High nutritional risk (NRS ≥ 3) was not statistically significant after adjustment (aOR = 1.7; 95% CI 0.5–6.2; *p* = 0.437), and major complications showed a non-significant trend toward higher risk (Clavien-Dindo ≥ III: aOR = 3.4; 95% CI 0.9–14.0; *p* = 0.085). Age (per +10 years) was not associated with the endpoint (aOR = 1.2; 95% CI 0.7–2.2; *p* = 0.483). Model discrimination was good (AUC = 0.81) with acceptable calibration (Hosmer-Lemeshow *p* = 0.47); however, because 42 composite events were modeled with several clinically relevant covariates, these adjusted estimates should be interpreted as exploratory rather than definitive (Table 6).

**Figure 1:**
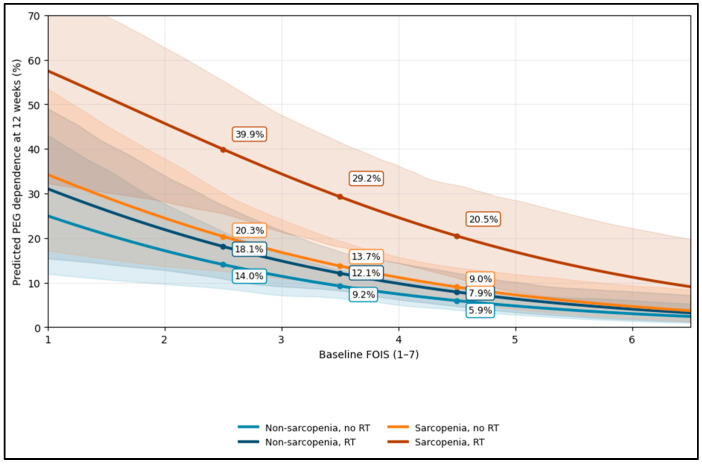
Exploratory PEG-risk curves; subgroup analysis by low skeletal muscle mass x radiotherapy.

In an exploratory multivariable model, predicted PEG risk fell as baseline FOIS increased, but it remained substantially higher in the low-skeletal-muscle-mass + radiotherapy stratum. For example, at FOIS 2.5, predicted PEG risk was 3.6% in no low skeletal muscle mass/no RT, 13.2% in no low skeletal muscle mass/RT, 13.2% in low skeletal muscle mass/no RT, and 38.7% in low skeletal muscle mass/RT. At FOIS 4.5, risks decreased to 1.1%, 4.5%, 4.5%, and 16.3%, respectively—showing that better baseline swallowing is protective, although the low-muscle-mass/RT subgroup retained higher residual risk (Figure 1).

**Figure 2:**
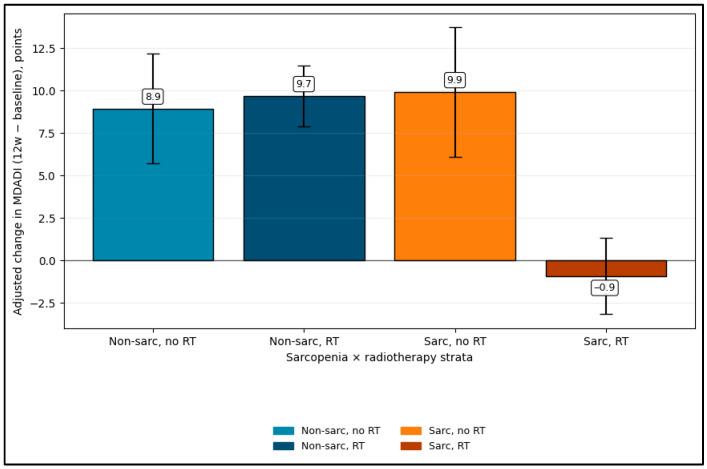
Adjusted DeltaMDADI; exploratory interaction model.

These findings demonstrate an exploratory low-skeletal-muscle-mass x radiotherapy interaction after adjustment for baseline MDADI, baseline FOIS, age, and major complications (interaction *p* = 0.0001). Adjusted mean an exploratory interaction after adjustment for baseline MDADI, baseline FOIS, age, and major complications (interaction *p* = 0.0001). Adjusted mean Delta MDADI was +8.9 points (95% CI 5.7 to 12.2) in no low skeletal muscle mass/no RT, +9.7 (95% CI 7.9 to 11.5) in no low skeletal muscle mass/RT, and +9.9 (95% CI 6.1 to 13.7) in low skeletal muscle mass/no RT. In contrast, the low skeletal muscle mass/RT subgroup showed minimal to no improvement, with adjusted Delta MDADI −0.9 points (95% CI −3.2 to 1.3), as seen in Figure 2. Given the small subgroup counts, this interaction should be viewed as hypothesis-generating rather than confirmatory.

**Figure 3:**
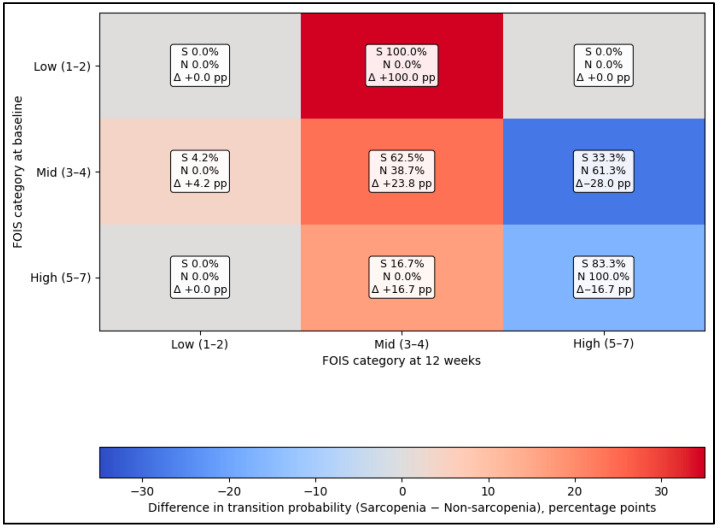
Difference heatmap of low-skeletal-muscle-mass-associated shifts in swallowing recovery trajectories. S means Sarcopenia; N means Non-sarcopenia; Δ, change score.

Figure 3 demonstrates that low skeletal muscle mass was associated with a less favorable pattern of swallowing recovery over 12 weeks, visualized as baseline-to-follow-up FOIS category transitions. Among patients starting in the mid FOIS category (3–4), those with low skeletal muscle mass were less likely to improve to high FOIS (5–7) compared with patients without low skeletal muscle mass (33.3% vs. 61.3%, Delta −28.0 percentage points) and were more likely to remain mid-to-mid (62.5% vs. 38.7%, Delta +23.8 pp), suggesting delayed or plateauing functional progress. Even among those beginning in the high FOIS group (5–7), patients with low skeletal muscle mass had a lower probability of staying high (83.3% vs. 100.0%, Delta −16.7 pp).

## 4. Discussion

### 4.1. Analysis of Findings

Across perioperative and early survivorship endpoints, the present cohort supports preoperative CT-defined low skeletal muscle mass as a clinically meaningful vulnerability state rather than a purely descriptive body-composition label. Patients with low skeletal muscle mass had higher frailty burden and a more inflammatory/low-protein biochemical profile at baseline, and this reduced physiologic reserve aligned with a more complicated early postoperative course. Biologically, this association is plausible because systemic catabolism, inflammation, and diminished skeletal-muscle reserve may extend to the suprahyoid and pharyngeal musculature, thereby reducing tolerance to postoperative disuse, impaired oral intake, and treatment-related tissue injury. We now avoid causal framing and interpret low skeletal muscle mass as a risk marker rather than a proven modifiable causal driver. This interpretation is consistent with prior head-and-neck surgery literature linking low muscle reserve to prolonged hospitalization and adverse postoperative recovery [16,17,18,19,20].

The most clinically salient differences emerged at 12 weeks, where patients with low skeletal muscle mass showed a less favorable functional-nutritional recovery profile: greater weight loss, lower FOIS scores, and substantially higher PEG dependence. In the adjuvant setting, radiotherapy-related mucosal injury can impair swallowing during the early postoperative period, while radiation-induced fibrosis and reduced tissue compliance may further compromise hyolaryngeal excursion and pharyngeal constriction over time [21,22,23]. Our 12-week window therefore likely captures only the early component of radiotherapy-associated swallowing burden, not the full late-fibrotic trajectory. This point is important when interpreting the higher poor-recovery odds observed with adjuvant radiotherapy in the current cohort.

Patient-reported outcomes add important interpretive depth to these functional endpoints. In this cohort, MDADI and global QoL improved in both groups, but gains were smaller in low skeletal muscle mass and were blunted in the low-muscle-mass/radiotherapy subgroup. The 12-week absolute MDADI gap of 9.5 points approaches the approximately 10-point between-group MCID proposed for head-and-neck cancer populations [24,25,26], supporting potential clinical relevance, whereas the smaller between-group difference in change scores should be interpreted more cautiously. Recent systematic surgical literature also supports contextualizing early FOIS-, PEG-, and MDADI-related recovery within a broader continuum of postoperative swallowing adaptation: Guarino et al. summarized contemporary speech and swallowing outcomes after TORS for oropharyngeal squamous cell carcinoma and highlighted substantial heterogeneity in early feeding-tube and dysphagia trajectories across surgical series [27]. This broader evidence base reinforces that our 12-week outcomes are clinically meaningful but should not be interpreted as the final functional endpoint.

The low-skeletal-muscle-mass and radiotherapy interaction is noteworthy, but it should be interpreted conservatively. Patients with reduced muscle reserve may have less capacity to buffer acute swallowing stress through compensatory biomechanics, effective cough, and tolerance of rehabilitation intensity. However, because subgroup counts were small and the study was not powered primarily for interaction testing, we interpret this signal as exploratory and hypothesis-generating rather than definitive evidence of synergy. Future studies with larger cohorts and longer follow-up should determine whether low skeletal muscle mass truly modifies the late fibrotic swallowing effects of adjuvant radiotherapy.

From an implementation standpoint, a major advantage of this framework is feasibility: CT imaging is already embedded in routine oncologic pathways, and cervical-level muscle metrics can be extracted without additional patient burden. Nevertheless, the present imaging approach should be interpreted as a pragmatic C3-based estimate of low skeletal muscle mass rather than a direct L3 gold-standard measurement or a full consensus sarcopenia diagnosis. In practice, incorporating this low-burden marker into perioperative assessment may help prioritize nutrition resources, swallowing surveillance, and rehabilitation intensity for patients at higher early risk, while awaiting larger studies that validate standardized thresholds and integrate strength/performance testing [20,28].

Because staging CT is already embedded in routine care, incorporating CT-defined low skeletal muscle mass into perioperative workflows may support earlier dietitian escalation, structured protein/energy targets, earlier swallow-therapy contact, and closer early follow-up. The added risk seen with adjuvant radiotherapy suggests that the combination of low skeletal muscle mass and planned RT may represent a higher-priority phenotype for intensified prehabilitation/rehabilitation pathways and anticipatory discharge planning. These are proposed clinical implications that require prospective validation rather than immediate causal inference.

These findings should be interpreted within the specific clinical context of this cohort. Although the results suggest that preoperative CT-defined low skeletal muscle mass is associated with less favorable early postoperative functional recovery, the observed associations may also reflect the influence of multiple interrelated clinical factors, including comorbidity burden, tumor site and extent, reconstructive complexity, baseline nutritional and functional status, PEG dependence, other patient factors and comorbidities, and adjuvant treatment exposure [29,30,31,32,33,34,35,36,37]. Accordingly, the findings are best understood as context-dependent associations that may help identify a more vulnerable postoperative subgroup, rather than as evidence of an isolated effect independent of the broader perioperative and oncologic profile.

### 4.2. Study Limitations

This single-center learning-oriented study had a modest sample size (*n* = 74), which limits precision for subgroup and interaction estimates and raises concern for model overfitting despite the use of clinically selected covariates. The imaging exposure captured low skeletal muscle mass only; muscle strength and physical performance—core components of consensus sarcopenia definitions—were not measured, so the full syndrome of sarcopenia cannot be diagnosed in this dataset. In addition, the CT metric was derived pragmatically at C3 rather than measured directly at L3, and the SMI threshold was cohort-internal rather than universally validated. Residual confounding remains possible, particularly because tumor site/subsite, extent of resection, type of free-flap reconstruction, PEG status at discharge, rehabilitation intensity, and clinician thresholds for PEG placement were not all modeled. We also relied on intake scales and patient-reported outcomes rather than standardized instrumental swallow studies such as videofluoroscopy or FEES, which limits physiologic interpretation. Finally, outcomes were limited to 12 weeks, so later radiotherapy-induced fibrosis, longer-term swallowing adaptation, and survivorship trajectories were not captured.

## 5. Conclusions

CT-defined low skeletal muscle mass was associated with worse early postoperative course and less favorable 12-week recovery, including greater weight loss, poorer oral intake, lower dysphagia-related quality of life, and higher PEG dependence. Low skeletal muscle mass remained associated with the composite poor-recovery endpoint after adjustment, but these findings should be interpreted as exploratory and hypothesis-generating. Larger multicenter studies incorporating consensus sarcopenia definitions, instrumental swallowing assessment, tumor-site granularity, and longer follow-up are needed before this imaging biomarker can be used as a definitive clinical decision tool.

## Figures and Tables

**Table 1:** Baseline characteristics by CT-defined low skeletal muscle mass.

Variable	Low Skeletal Muscle Mass (*n* = 32)	No Low Skeletal Muscle Mass (*n* = 42)	*p*
Age, years	62.0 ± 9.1	56.9 ± 9.3	0.016
Male sex, *n* (%)	23 (71.9)	31 (73.8)	0.854
BMI, kg/m^2^	24.1 ± 3.4	27.9 ± 4.0	<0.001
NRS-2002 score	3.6 ± 0.8	2.9 ± 0.9	<0.001
mFI-5 frailty score (0–5)	1.6 ± 1.0	1.2 ± 0.8	0.02
Albumin, g/dL	3.3 ± 0.4	3.8 ± 0.4	<0.001
CRP, mg/L	18.4 ± 10.8	12.7 ± 9.8	0.019
Skeletal muscle index, cm^2^/m^2^	38.8 ± 3.3	47.3 ± 4.0	<0.001
Baseline FOIS (1–7)	3.4 ± 1.0	3.8 ± 1.1	0.118
MDADI composite baseline (0–100)	53.4 ± 10.3	57.9 ± 11.9	0.088
Current smoker, *n* (%)	11 (34.4)	14 (33.3)	0.921
Advanced stage (III–IV), *n* (%)	21 (65.6)	24 (57.1)	0.464
Planned adjuvant radiotherapy, *n* (%)	26 (81.2)	31 (73.8)	0.453

BMI, body mass index; CRP, C-reactive protein; FOIS, Functional Oral Intake Scale; MDADI, MD Anderson Dysphagia Inventory; mFI-5, 5-item modified Frailty Index; NRS-2002, Nutritional Risk Screening 2002.

**Table 2:** Perioperative course and 30-day outcomes by low skeletal muscle mass.

Outcome	Low Skeletal Muscle Mass (*n* = 32)	No Low Skeletal Muscle Mass (*n* = 42)	*p*
Free-flap reconstruction, *n* (%)	18 (56.2)	26 (61.9)	0.622
Operative time, hours	7.8 ± 1.2	7.3 ± 1.3	0.138
ICU stay, days	2.1 ± 1.1	1.7 ± 0.9	0.075
Length of stay, days	13.4 ± 4.1	10.3 ± 3.3	<0.001
PEG at discharge, *n* (%)	19 (59.4)	16 (38.1)	0.072
Tracheostomy at discharge, *n* (%)	14 (43.8)	14 (33.3)	0.363
Any 30-day complication, *n* (%)	15 (46.9)	15 (35.7)	0.332
Major complication (Clavien–Dindo ≥ III), *n* (%)	10 (31.2)	5 (11.9)	0.04
Unplanned reoperation ≤ 14 days, *n* (%)	7 (21.9)	5 (11.9)	0.244
Flap compromise/loss, *n* (%)	2 (6.2)	1 (2.4)	0.575
30-day readmission, *n* (%)	4 (12.5)	6 (14.3)	0.823

ICU, intensive care unit; PEG, percutaneous endoscopic gastrostomy.

**Table 3:** Twelve-week recovery outcomes by low skeletal muscle mass.

Outcome	Low Skeletal Muscle Mass (*n* = 32)	No Low Skeletal Muscle Mass (*n* = 42)	*p*
Weight loss from baseline, %	10.9 ± 3.4	8.6 ± 2.6	0.003
FOIS at 12 weeks (1–7)	3.9 ± 1.1	4.6 ± 1.1	0.01
FOIS change (12 w − baseline)	0.5 ± 0.8	0.8 ± 0.8	0.1
MDADI composite at 12 weeks (0–100)	57.1 ± 10.9	66.6 ± 11.9	0.001
EORTC QLQ-C30 global health at 12 weeks (0–100)	60.4 ± 10.6	66.3 ± 11.0	0.022
EORTC QLQ-H&N35 swallowing at 12 weeks (0–100; higher = worse)	47.2 ± 14.4	38.8 ± 15.8	0.021
HADS-Anxiety at 12 weeks (0–21)	9.3 ± 3.2	7.8 ± 3.2	0.05
HADS-Depression at 12 weeks (0–21)	8.8 ± 3.2	7.2 ± 3.1	0.032
PEG dependence at 12 weeks, *n* (%)	10 (31.2)	4 (9.5)	0.018
Tracheostomy dependence at 12 weeks, *n* (%)	5 (15.6)	3 (7.1)	0.281
Poor recovery composite *n* (%)	24 (75.0)	18 (42.9)	0.006

EORTC QLQ-C30, European Organisation for Research and Treatment of Cancer Quality of Life Questionnaire-Core 30; EORTC QLQ-H&N35, EORTC Head and Neck cancer module; FOIS, Functional Oral Intake Scale; HADS, Hospital Anxiety and Depression Scale; MDADI, MD Anderson Dysphagia Inventory; PEG, percutaneous endoscopic gastrostomy; 12 w, 12 weeks.

**Table 4:** Patient-reported outcome trajectories (baseline to 12 weeks), by low skeletal muscle mass.

Measure	Low Skeletal Muscle Mass Baseline	Low Skeletal Muscle Mass 12 w	Low Skeletal Muscle Mass Delta (12 w-Baseline)	No Low Skeletal Muscle Mass Baseline	No Low Skeletal Muscle Mass 12 w	No Low Skeletal Muscle Mass Delta (12 w-Baseline)	*p* (Within Low Muscle)	*p* (Within No Low Muscle)	*p* (Delta Difference)
MDADI composite (0–100; higher = better)	53.4 ± 10.3	57.1 ± 10.9	3.6 ± 6.4	57.9 ± 11.9	66.6 ± 11.9	8.7 ± 6.4	0.002	<0.001	0.045
EORTC QLQ-C30 Global Health (0–100; higher = better)	50.9 ± 11.3	60.4 ± 10.6	9.6 ± 9.4	54.3 ± 12.9	66.3 ± 11.0	11.9 ± 9.2	<0.001	<0.001	0.023
EORTC QLQ-H&N35 Swallowing (0–100; higher = worse)	54.5 ± 13.1	47.2 ± 14.4	−7.3 ± 10.6	47.3 ± 15.7	38.8 ± 15.8	−8.6 ± 11.7	<0.001	<0.001	0.58
HADS-Anxiety (0–21)	10.3 ± 3.0	9.3 ± 3.2	−1.0 ± 2.3	9.6 ± 3.3	7.8 ± 3.2	−1.8 ± 2.2	0.015	<0.001	0.048
HADS-Depression (0–21)	9.6 ± 3.2	8.8 ± 3.2	−0.8 ± 2.2	8.9 ± 3.3	7.2 ± 3.1	−1.7 ± 2.2	0.036	<0.001	0.032

Delta, change (12 weeks − baseline); EORTC QLQ-C30, European Organisation for Research and Treatment of Cancer Quality of Life Questionnaire-Core 30; EORTC QLQ-H&N35, EORTC Head and Neck cancer module; HADS, Hospital Anxiety and Depression Scale; MDADI, MD Anderson Dysphagia Inventory; 12 w, 12 weeks.

**Table 5:** Correlations among 12-week change scores (Δ).

Variable 1	Variable 2	r	*p*
ΔMDADI	ΔEORTC Global Health	0.48	<0.001
ΔMDADI	ΔH&N35 Swallowing	−0.42	<0.001
ΔFOIS	ΔMDADI	0.36	0.002
ΔWeight loss %	ΔEORTC Global Health	−0.31	0.008
ΔHADS-Depression	ΔMDADI	−0.4	<0.001
Baseline NRS	Weight loss % at 12 w	0.34	0.003

Δ, change score; EORTC QLQ-C30, European Organisation for Research and Treatment of Cancer Quality of Life Questionnaire-Core 30; EORTC QLQ-H&N35, EORTC Head and Neck cancer module; FOIS, Functional Oral Intake Scale; HADS, Hospital Anxiety and Depression Scale; MDADI, MD Anderson Dysphagia Inventory; NRS, Nutritional Risk Screening.

**Table 6:** Multivariable logistic regression exploring poor recovery at 12 weeks.

Predictor	aOR	95% CI	*p*
CT-defined low skeletal muscle mass (yes)	5.4	1.4–24.0	0.016
High nutritional risk (NRS ≥ 3)	1.7	0.5–6.2	0.437
Major complication (Clavien–Dindo ≥ III)	3.4	0.9–14.0	0.085
Adjuvant radiotherapy (yes)	4.3	1.0–24.0	0.049
Baseline FOIS (per +1 point)	0.6	0.4–0.9	0.02
Age (per +10 years)	1.2	0.7–2.2	0.483

Outcome: Poor recovery composite (PEG at 12 weeks and/or FOIS ≤ 3 and/or MDADI < 55). MDADI < 55 was used as an empirically chosen early postoperative threshold rather than a validated diagnostic cut-off. Model performance; Hosmer-Lemeshow *p* = 0.47; aOR, adjusted odds ratio; AUC, area under the receiver operating characteristic curve = 0.81; CI, confidence interval; CT, computed tomography; FOIS, Functional Oral Intake Scale; NRS, Nutritional Risk Screening.

## Data Availability

The data presented in this study are available on request from the corresponding author.
